# Characterizing stomatal attributes and photosynthetic induction in relation to biochemical changes in *Coriandrum sativum* L. by foliar-applied zinc oxide nanoparticles under drought conditions

**DOI:** 10.3389/fpls.2022.1079283

**Published:** 2023-01-12

**Authors:** Shakil Ahmed, Muhammad Tajammal Khan, Asim Abbasi, Inzamam Ul Haq, Aiman Hina, Muhammad Mohiuddin, Muhammad Atiq Ur Rehman Tariq, Muhammad Zaheer Afzal, Qamar uz Zaman, Anne Wai Man Ng, Yong Li

**Affiliations:** ^1^ Institute of Botany, University of the Punjab Quaid, Lahore, Pakistan; ^2^ Division of Science and Technology, Department of Botany, University of Education, Lahore, Pakistan; ^3^ Department of Environmental Sciences, Kohsar University, Murree, Pakistan; ^4^ College of Plant Protection, Gansu Agricultural University, Lanzhou, China; ^5^ Department of Botany, Kohsar University, Murree, Pakistan; ^6^ Department of Environmental Sciences, Comsats University Islamabad (CUI), Abbottabad, Pakistan; ^7^ Center of Excellence in Water Resources Engineering, University of Engineering and Technology, Lahore, Pakistan; ^8^ Department of Environmental Sciences, University of Jhang, Jhang, Pakistan; ^9^ Department of Environmental Sciences, The University of Lahore, Lahore, Pakistan; ^10^ College of Engineering, IT and Environment, Charles Darwin University, Darwin, NT, Australia; ^11^ National Engineering Laboratory for Applied Forest Ecological Technology in Southern China, Faculty of Life Science and Technology, Central South University of Forestry and Technology, Changsha, China

**Keywords:** abscisic acid, biological yield, micrograph, net photosynthesis, phenolic content, principal component analysis, stomatal conductance, stomatal density

## Abstract

Abiotic stress, particularly drought, will remain an alarming challenge for sustainable agriculture. New approaches have been opted, such as nanoparticles (NPs), to reduce the negative impact of drought stress and lessen the use of synthetic fertilizers and pesticides that are an inevitable problem these days. The application of zinc oxide nanoparticles (ZnO NPs) has been recognized as an effective strategy to enhance plant growth and crop production during abiotic stress. The aim of the current study was to investigate the role of ZnO NPs in drought stress management of drought-susceptible *Coriandrum sativum* L. (*C. sativum*) in two consecutive seasons. Drought regimes (moderate drought regime—MDR and intensive drought regime—IDR) were developed based on replenishment method with respect to 50% field capacity of fully irrigated (control) plants. The results showed that foliar application of 100 ppm ZnO NPs improved the net photosynthesis (Pn), stomatal conductance (*C*), and transpiration rate (*E*) and boosted up the photosynthetic capacity associated with photosynthetic active radiation in MDR. Similarly, 48% to 30% improvement of chlorophyll b content was observed in MDR and onefold to 41% in IDR during both seasons in ZnO NP-supplemented plants. The amount of abscisic acid in leaves showed a decreasing trend in MDR and IDR in the first season (40% and 30%) and the second season (49% and 33%) compared with untreated ZnO NP plants. The ZnO NP-treated plants showed an increment in total soluble sugars, total phenolic content, and total flavonoid content in both drought regimes, whereas the abaxial surface showed high stomatal density and stomatal index than the adaxial surface in foliar-supplied NP plants. Furthermore, ZnO NPs improve the magnitude of stomata ultrastructures like stomatal length, stomatal width, and pore length for better adaptation against drought. Principal component analysis revealed the efficacy of ZnO NPs in inducing drought tolerance in moderate and intensive stress regimes. These results suggest that 100 ppm ZnO NPs can be used to ameliorate drought tolerance in *C. sativum* plants.

## 1 Introduction

Drought is one of the critical environmental factors impairing plants’ metabolic machinery, thereby restricting the growth, development, and overall agricultural yield globally (Tanveer et al., 2019). Major aspects of drought conditions are global warming, changes in the pattern of precipitation, and limited access to underground water. Mostly, drought affects physio-biochemical processes like accumulation of osmo-protectants, reduction in phenolic and flavonoid content, and production of reactive oxygen species (Afshari et al., 2021; Srivastava et al., 2021). Water deficit condition restrains the efficacy of photosynthetic activity, causes significant damage to the ultrastructure of cells, and distorts the metabolic process (Liu et al., 2022b). Furthermore, water deficiency affects the integrity of thylakoid membranes, accelerates leaf senescence, induces necrosis and chlorosis, and degrades the chlorophyll content (Demmig-Adams et al., 2018; Desoky et al., 2021; Batool et al., 2022). Therefore, plants have adapted to ensure their survival under drought stress through osmotic adjustment, activation of the antioxidant defense system, and modulation of stomatal attributes (Sharma et al., 2020).

Plants show the most prominent response through stomatal regulations against climatic variability, particularly drought (Song et al., 2022). Both abaxial (lower surface of leaf) and adaxial (upper surface of leaf) stomata are important gateways for the exchange of gases (Wankmüller and Carminati, 2022). Stomatal density and stomatal index are the key features concerning the determinants of photosynthesis, transpiration rate, and stomatal conductance (Lei et al., 2018). A number of reports that are associated with the response of stomatal attributes on account of salinity, extreme temperature, canopy cover and plant density, elevated carbon dioxide, variation in active photosynthetic radiations, and insufficient water availability are available (Arve et al., 2011; Bharath et al., 2021; Li et al., 2021; Ramos-Zambrano et al., 2021; Abdalla et al., 2022).

Stomatal regulation is one of the features that decide the fate of plant growth, development, and yield through photosynthetic activity. Photosynthesis is a driving force of various physiological and biochemical processes (Verma et al., 2020). Under drought stress, the internal concentration of CO_2_ is decreased due to stomatal closure, which changes the activities of enzymes, reduces ATP formation, disrupts the stability of the membrane, affects the regeneration of RuBP, limits the RUBISCO enzyme activity, and disturbs the overall phenomena of photosynthesis (Muhammad et al., 2021). Moreover, water stress disrupts the electron transport chain, reduces photosynthetic pigments, intensifies photo-inhibition, decreases the water potential, and increases turgor pressure to impair plant growth (Chen et al., 2017; Semerci et al., 2017; Li et al., 2019).

In addition, an important phytohormone that plays a vital part in drought stress response is abscisic acid (ABA), which is involved in stomatal operations (opening and closing) through biochemical signal transduction (Müller, 2021). It acts as a primary mediator in water deficit conditions and confers plant drought tolerance (Verma et al., 2016). Therefore, ABA has mainly been accompanied by regulating water deficit conditions in plants (Nian et al., 2021)—for instance, the endogenous level of ABA increased exponentially in *Arabidopsis*, wheat, rice, tomato, soybean, maize, and sesame under drought conditions (Dossa et al., 2020). It has been documented that ABA indirectly regulates plant development by modifying stomatal resistance to control transpiration and CO_2_ uptake and control the eco-physiological features of the plant (Zhang et al., 2012; Dong et al., 2017; Liang et al., 2022). Furthermore, ABA is a regulatory component in seed germination, biomolecule synthesis, senescence, and root architecture modification under water deficit conditions (Liu et al., 2022a). These adaptive changes induced by ABA may be of significant importance for the survival and better growth of *Coriandrum sativum* with limited or no water supply. Therefore, these findings indicated that ABA-assisted drought tolerance is a prerequisite for plants to respond fully to drought.

The provision of microelement supplements like Co, Cu, Mn, Ni, Fe, and Zn has proven to boost crop production, particularly under drought conditions (Ashraf et al., 2012; Tripathi et al., 2018). Among them, Zn plays a very vital role in amelioration of drought tolerance (Farooq et al., 2021). Zn improves the performance of plants through the regulation of various processes like overexpression of proteins to stimulate the defensive mechanism and accumulation of osmo-protectants. Furthermore, it increases the germination index, significantly improves cell division, improves water use efficiency and plant water relation, and interacts with plant growth regulator to cope up the drought effects (Umair Hassan et al., 2020). It is also involved in carbon metabolism due to the integral component of carbonic anhydrase, biomolecules such as phospholipids, and co-factor of auxins, activator of adolase, and therefore it plays an important role in plant nucleic acid metabolism (Umair Hassan et al., 2020).

However, foliar application of nanoparticles (NPs) as nanofertilizers and growth regulator is becoming more popular in agriculture compared with the typical soil–root treatment. It is due to the stomata which are primarily the entry source for NPs and easily transported to other plant sections through apoplastic and symplastic routes. Therefore, foliar application of green synthesized engineered NPs enhances the efficiency of plant growth as well as plant protection strategies (Hong et al., 2021).

A limited number of studies have shown that the appropriate application of ZnO NPs can regulate drought tolerance in different crops as wheat, sunflower, tomato, and red cabbage (Umair Hassan et al., 2020). However, there is lack of information regarding ZnO NP-induced mechanisms conferring drought tolerance in plants. Therefore, the current study addressed the impact of ZnO NPs in physiological, biochemical, and stomatal attributes to induce drought stress tolerance and to evaluate the role of stress hormone (ABA) in the induction of drought tolerance under different water regimes. Therefore, changes induced by osmoregulators on account of ZnO NP application may be of significant importance for the survival and better growth of *C. sativum* with limited water supply. Therefore, it assumed that ZnO NPs may have an ameliorative role in drought tolerance against different irrigation regimes.

## 2 Materials and methods

### 2.1 Experimental location and climatic data

Experiment was conducted in the research area of the Department of Botany, University of Education Lahore (Dera Ghazi Khan Campus) during the period 2019/2020 and 2020/21 from early November to late March under natural conditions. The site specification was 30°06′ N (longitude) and 70°62′ E (latitude) at an elevation of 129 m above sea level. The climate of this area is subtropical dry arid with 104 mm average annual rainfall. The average temperature from November to April was 25 ± 3°C and 23± 1°C in two consecutive seasons. The total annual precipitation was 173 and 104 mm in both seasons, respectively.

### 2.2 Properties of irrigated water and soil

The physio-chemical properties of water and soil (obtained from botanical garden; 30.067423, 70.627243) are given in **Tables 1**, **2**. All plastic pots (25 cm × 20 cm × 15 cm) were filled with 7.2 kg of clay-loamy-textured soil with a leaching hole at the bottom.

**Table 1:** Chemical properties of irrigation water.

EC(dS/m)	Cations(mEq/L)	Anions(mEq/L)	Na+(mEq/L)	Ca++(mEq/L)	Mg++(mEq/L)	Cl^-^(mEq/L)	SO2(mEq/L)	TDS(ppm)	$\text{C}{\text{O}}_{3}^{-}$ (mEq/L)	$\text{HC}{\text{O}}_{3}^{-}$ (mEq/L)	SAR	pH
0.4	8.6	8.6	1.29	3.7	2.1	1.13	1.02	256	0	2.17	0.98	7.11

EC, Electrical conductivity.

**Table 2:** Physiochemical analysis of soil.

EC(dS/m)	Cations(mEq/L)	Anions(mEq/L)	Na+(mEq/L)	Ca++(mEq/L)	Mg++(mEq/L)	Cl^-^(mEq/L)	SO2(mEq/L)	TDS(ppm)	$\text{C}{\text{O}}_{3}^{-}$ (mEq/L)	$\text{HC}{\text{O}}_{3}^{-}$ (mEq/L)	SAR	pH
0.4	8.6	8.6	1.29	3.7	2.1	1.13	1.02	256	0	2.17	0.98	7.11

EC, Electrical conductivity.

### 2.3 Growth conditions and experimental design

The *C. sativum* var Dilpazeer (lot number FD-898602) seeds were obtained from Vegetable Research Institute, Faisalabad, Pakistan. The seeds (sterilized) were sown in the second week of November in both seasons, with a density of 10 per plastic pot and maintained to five plants/pot until seedling establishment (Se). At each time, the pots were watered to their respective field capacity. The pots were placed under natural conditions to match the almost field conditions. The weather forecast was monitored after every 4 h to make necessary arrangements before the account of rain ZnO NPs. Therefore, rain protected sheet (transparent) was used to prevent the experiment from the effect of precipitation.

Drought stress applied after the seedling establishment (after 24 days) based on keeping the water content at field capacity (FC) of soil. It was calculated by flooding 7.2 kg potted test soil without plants and allowing the water to trench off completely for 12 h and weighed as initial weight (Iw). Dried this soil at 105 °C for 24 hours in oven and weighed it as final weight (Fw). Thereafter, the FC % was calculated by the following formula, namely, Equation 1:


FC % = (Iw - Fw/Fw) *100

The benchmark for the next irrigation was 50% of FC of control or fully irrigated (FI). Therefore, on set of 50% FC in FI, there was application of 75% and 50% irrigation (with respect to 50% FC of FI) to generate drought stress regimes (MDR—moderate drought regime and IDR—intensive drought regime) as mentioned in Appendix A. Therefore, inspection of reduction of water from field capacity was conducted throughout the season by usual soil sampling on a weekly basis through gravimetric method (105°C, 24 h). The same protocol was followed for replenishment throughout the experiment until harvest. Drought stress was applied on December 9, 2019 and December 15, 2020 after seedling establishment.

Zinc oxide nanoparticles (ZnO NPs) of average size 37 nm were obtained from Applied Environment Biology & Environmental Biotechnology Research Lab, Institute of Botany, University of Punjab, Lahore, Pakistan. This article is an extension of our previous research paper in which the optical specifications of NPs were described (Khan et al., 2021). Foliar application of ZnO NPs was initiated from the 10th day of applying drought stress and continued at regular interval of 10 days for three times (2 ml for each pot every time).

In this experiment, the influence of three irrigation regimes, including two drought regimes MDR and IDR, was evaluated through eco-physiological, bio-chemical, and stomatal analysis, and its adaptive response to drought stress under foliar-applied ZnO NP *C. sativum* was examined. This experiment was designed to determine the performance of FI, MDR, and IDR along with the efficacy of ZnO NPS (control; 0 and 100 ppm). In previous studies, 100 ppm ZnO NPs showed a strong effect on *C. sativum* growth attributes in pot conditions (Stakhova et al., 2000; Khan et al., 2021). Therefore, the following six treatments were designed based on previous trials:

**Table:** 

Treatment	Description
TR1	FI
TR2	FI + ZnO NPs (100 ppm)
TR3	MDR
TR4	MDR + ZnO NPs (100 ppm)
TR5	IDR (intensive drought regime)
TR6	IDR + ZnO NPS (100 ppm)

where TR is treatment, FI is full irrigation, MDR is moderate drought regime, IDR is intensive drought regime, and ZnO NPs is zinc oxide nanoparticles.

### 2.4 Assessment of eco-physiological features

Photosynthetic rate (Pn_)_, transpiration rate (*E*), and stomatal conductance (*C*) were used to measure, from two healthy and totally expanded leaves of the main stem at constant vapor pressure, deficit of about 2.5 kPa (0.08 ± SE) with ambient air temperature of the leaf chamber at 25.0 ± 0.18°C through infrared gas analyzer (CI-340 Portable Photosynthesis System; CID Bio-Science Inc., Washington, USA) (Mitchell, 1992). These parameters were examined from 9:30 am to 11:30 am. Sunlight was used as the light source by customizing the quantum flux of the photosynthesis system according to sunny situation.

### 2.5 Measurement of chlorophyll and ABA content

Chlorophyll a and b content (mg/g), respectively, was calculated using the method of Arnon (1949) and Davies and Goodwin (1976). In detail, 1 g of finely diced fresh leaves was crushed with a solution of one part 0.1 normal (N) ammonium hydroxide solution to nine parts acetone v/v (volume to volume). Then, it was centrifuged for 5 min at 5,000–10,000 rpm, and the supernatant was diluted to a quantity that produces an absorbance value of between 0.2 and 0.8 at wavelengths of 663 and 645 nm (Spectro UV-Vis 2505, Labomed, Los Angeles, CA, USA). The absorbance of each solution was measured by comparing with a blank solvent, and the content of chlorophyll a and b, respectively, was determined.

ABA content was determined through the ABA immunological bioassay test kit of Agresera AS20 4392 (ELISA) by following the catalog number CSB-E09159P. A total of 1 mg of fresh leaves of coriander was ground with 1 ml of lysis buffer and placed on ice for 1.5 h. Afterwards, a homogenized mixture was obtained with the help of a homogenizer (Bead Bug, Bead Mill Homogenizer; Benchmark Scientific, NJ, USA). Then, the mixture was centrifuged at 12,000 rpm for 4 m. Dilutions were prepared at 0, 0.155, 0.312, 0.625, 1.25, 2.5, and 5 µg/ml for standard curve calibration. Then, 50 µl of dilutions and samples was added into microplate wells (pre-coated with ABA-specific antibody), followed by the addition of horseradish peroxidase (HRP) conjugate (50 µl) in each well except blank. Incubation was done at 37°C for 1 h. A competitive inhibition reaction with pre-coated antibodies was initiated between labeled ABA (HRP) and unlabeled ABA. Then, 100 µl of substrate was added in the dark (to prevent temperature fluctuations) to each well. Incubation was done again at 37°C for 15 min. The reaction was stopped by using 50 µl of stop solution in each well. The optical absorbance of each well was observed at 450 nm by using a microplate reader (DR-200Bs Microplate Reader, Hangzhou Boyn Instrument Co., Hangzhou, China).

### 2.6 Quantification of total soluble sugar, phenolic, and flavonoid production

Total soluble sugar (TSS) was measured by following the method of Jayaraman (1981). Specifically, 0.5 g of fresh leaves was taken and ground into liquid nitrogen along with 5 ml of ethanol (95%) to discharge out sugar, and then 5 ml of ethanol (75%) was added. The mixture was centrifuged at 4,000 rpm for 15 min. This solution was kept in the refrigerator for 1 week at below 4°C. The fresh anthrone reagent was prepared by adding 150 mg of anthrone into 100 ml of H_2_SO_4_ (72%). Then, 0.1 ml of stored ethanolic extract was prepared, mixed with anthrone reagent of 3 ml, and put into a water bath at 95°C. A UV spectrophotometer was used to measure the absorbance at 625 nm.

Dried leaves (10 g) from each treatment were obtained for the preparation of leaf extract. The samples were added with 75 ml of ethanol (95% v/v) for 10 min at 40°C. This extraction method was repeated three times. The extract was heated to evaporate the solvent at 40°C. The dried extract was used for further analysis. About 20–50 mg of the dried extract was added in 5 ml methanol and sonicated for 45 min (Ultra sonicated bath, Branson 2510, Marshal Scientific, NH, USA) at 40°C, followed by centrifugation for 10 min at 1,200 rpm. A clear supernatant was obtained and stored in amber glass bottle for further analysis.

Singleton and Rossi (1965) measured the total phenolics content (TPC) of the leaf extract by using the Folin–Ciocalteu reagent with some modifications. Sample and standard curve readings were measured through a spectrophotometer at 765 nm against the blank (reagent). The given sample (0.2 ml) was mixed well with the Folin–Ciocalteu reagent (0.2 ml) and added with water (0.6 ml) by 1:1. After 5–8 min had passed, a saturated solution of NaCO_3_ (8% w/v) was prepared. Then, 1 ml of saturated solution was taken and supplemented into the mixture. Moreover, distilled water was used to adjust the volume to up to 3 ml. This reaction combination was placed in the dark for 35 min. Afterwards, the solution was centrifuged for 8 min at 4,000 rpm. The absorbance peaks of the supernatant (blue color) were observed at 765 nm. The TPC was measured as gallic acid equivalent (GA Eq)/gram dry weight (g d. wt.) with respect to the standard calibration curve of GA (0 to 100 mg/ml).

Total flavonoid content (TFC) was measured through the aluminum colorimetric method (Meda et al., 2005). For the determination of TFC, the standard calibration curve of quercetin (C_15_H_10_O_7_) was used. A stock solution of quercetin was formed by adding 5 mg C_15_H_10_O_7_ in 1 ml methanol (CH_4_OH). Then, the standard concentrations of C_15_H_10_O_7_ (0.25 to 1 mg/ml) were prepared by serial dilutions using CH_4_OH. Then, 1 ml of each concentration was taken and put into a test tube, and distilled water (4 ml) was poured into the test tube. In addition, 5% of NaNO_2_ (0.3 ml) and 10% of AlCl_3_ (0.3_ ml_) water were put into test tubes after 5 min. Then, 1 M of NaOH (2 ml) was added after 5 min, and the volume was increased to 10 ml by adding distilled water. After stirring, the mixture was placed for 60 min at 25°C. A UV spectrophotometer (Labomed Spectro UV-2505) was used to calculate the absorbance at 510 nm against blank. The amount of TFC in the test samples was estimated through the standard curve and expressed as milligram quercetin equivalent (QE)/g of dried weight.

### 2.7 Evaluation of stomatal micrographs

Stomatal index (SI) was calculated using the approach of Salisbury (1928) based on an average of 50 microscopic fields by using the given formula:


(2) SI = S × 100/E + S

where S was the number of stomata/unit area, and *E* was the number of epidermal cells within the same unit area. Stomatal density (SD_dens_) was measured by using BM13070131 Biological Microscope (California, USA) at ×400 total magnification with a field of view 0.045 mm, followed by microscopic image processing with image J application (https://Imagej.nih.gov/ij/). The number of stomata was counted and divided by area of field-of-view to obtain the amount of stomata per millimeter square of leaf (Eisele et al., 2016). The option “sharpen” was used in ImageJ to better view the guard cells. The width and the length of the stomata opening were measured at 50-µm scale.

### 2.8 Statistical analysis and assessment

In the current study, a two factorial-based, completely randomized block design was used to sort out the experimental data. Data for each treatment were analyzed statistically by using STATISTICS 8.1 and MS Excel 2019, and the values were reported as means of four replications with standard error for difference in the means (SE) by following the analysis of variance (ANOVA) technique. The LSD test was then applied at probability *P<*0.05 to treatment means for ranking and comparison. Principal component analysis was done through Origin 8 Pro.

## 3 Results

### 3.1 Evaluation of photosynthetic response

The gaseous exchange parameters like photosynthetic active radiation (PAR), transpiration rate (*E*), stomatal conductance (C), internal carbon dioxide (Int. CO_2_), and net photosynthetic rate (Pn) were measured after post-anthesis stage to evaluate the effect ZnO NPs (100 ppm) under two regimes of drought MDR and IDR and well-watered (FI) plants.

**Figure 1:**
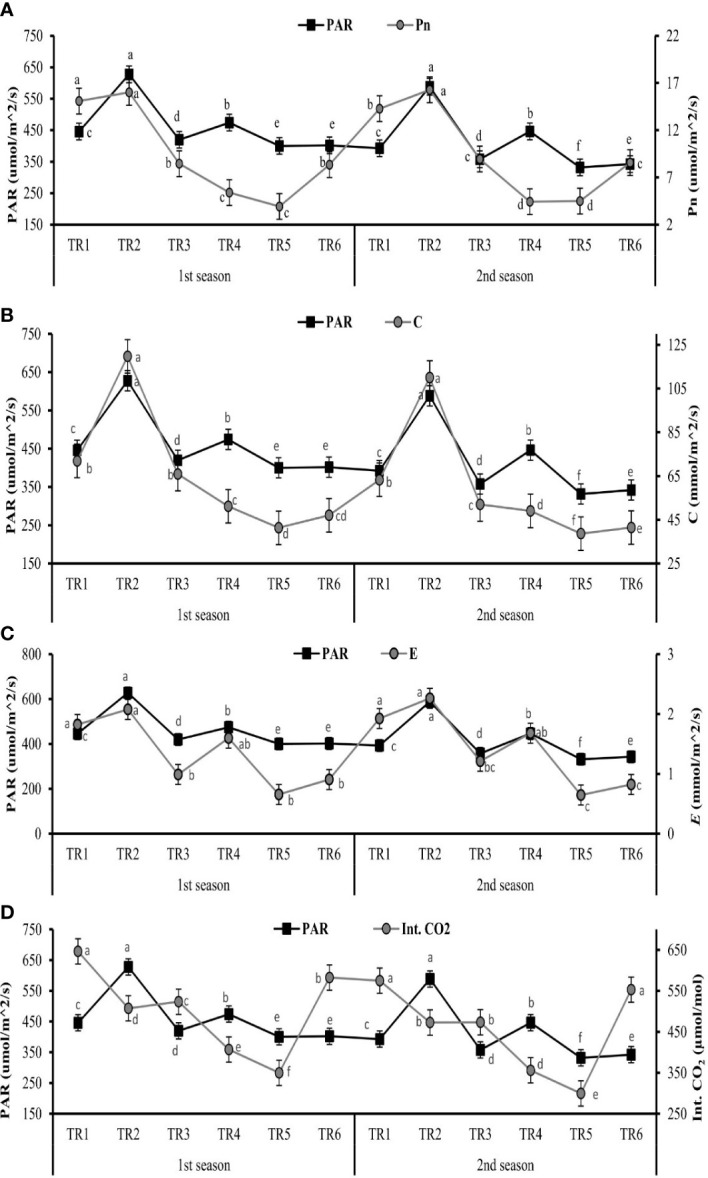
Effect of different treatments on **(A)** Pn (net photosynthesis), **(B)** C (stomatal conductance), **(C)** E (transpiration rate), and **(D)** Int. CO2 (internal carbon dioxide) in leaves and impact of photosynthetic active radiation on these physiological features of C. sativum after anthesis under drought regimes. The graph values are the mean ± SE of four replicates. Bars exhibited with different alphabets indicate the significant difference between samples by least significant difference (p ≤ 0.05). TR1: FI, TR2: FI + ZnO nanoparticles (NPs), TR3: MDR, TRT4: MDR + ZnO NPs, TRT5: IDR, and T6: IDR + ZnO NPs.

The values of PAR are drastically decreased after the anthesis stage. Significantly, the range of PAR was 627.65 to 400.25 µm/m^2^/s in the first season, while in the second season, this range was 588.27 to 331.72 µm/m^2^/s as shown in **Figure 1**.

The considerable increase in Pn was observed in TR2 (16.00 and 16.28 µm/m^2^/s) at the maximum value of PAR, when FI plants were treated with and ZnO NPs in both seasons as indicated in **Figure 1**. Under IDR, the maximum Pn was observed in TR6 (8.35 and 8.56 µm/m^2^/s) when the plants were treated with ZnO NPs, and the PAR value was 401.92 and 342.25 µm/m^2^/s for both seasons. The IDR plants (TR5) showed the lowest Pn when the least PAR was found, as indicated in **Figure 1A**. 

The optimum stomatal conductance was observed in TR2 (119.73 and 110.06 mmol/m^2^/s) in FI when treated with ZnO NPs, followed by MDR plants (51.19 and 49.5 mmol/m^2^/s) when PAR showed optimum values, as shown in **Figure 1B**, in the first and the second seasons. The TR5 plants showed the lowest C (41.35 and 38.67 mmol/m^2^/s) under IDR when plants were not treated with ZnO NPs in both consecutive seasons.

The highest *E* values—2.07 and 2.26 mmol/m^2^/s—were found in FI plants when treated with ZnO NPs indicated in TR2 followed by TR4 plants, as presented in **Figure 1C**, in both seasons when optimum PAR was present. The lowest *E* rate followed the same trend as those of Pn and C when the lowest PAR was present in TR5 plants in both seasons.

The amount of Int. CO_2_ in leaves showed an inverse relationship associated with PAR in FI and MDR in both seasons except IDR. The greater amount of Int. CO_2_ in the leaf of *C. sativum* was found in TR6 (582.75 and 552.32 µmol/mol) in the first and the second season when IDR plants were treated with ZnO NPs as indicated in **Figure 1D**, while the minimum amount of Int. CO_2_ was in TR5 (349.65 and 299.36 µmol/mol) in two consecutive seasons with respect to the lower values of PAR.

These results suggested that PAR plays a very vital role in photosynthetic induction. The highest value of PAR along with foliar application of ZnO NPs increased the Pn, *C*, and *E*, while it decreased the internal concentration of CO_2_ in FI and MDR, showing a contradictory association. In addition, the lowest PAR improves the Int. CO_2_ in IDR plants treated with ZnO NPs.

### 3.2 Determination of chlorophyll content

**Figure 2:**
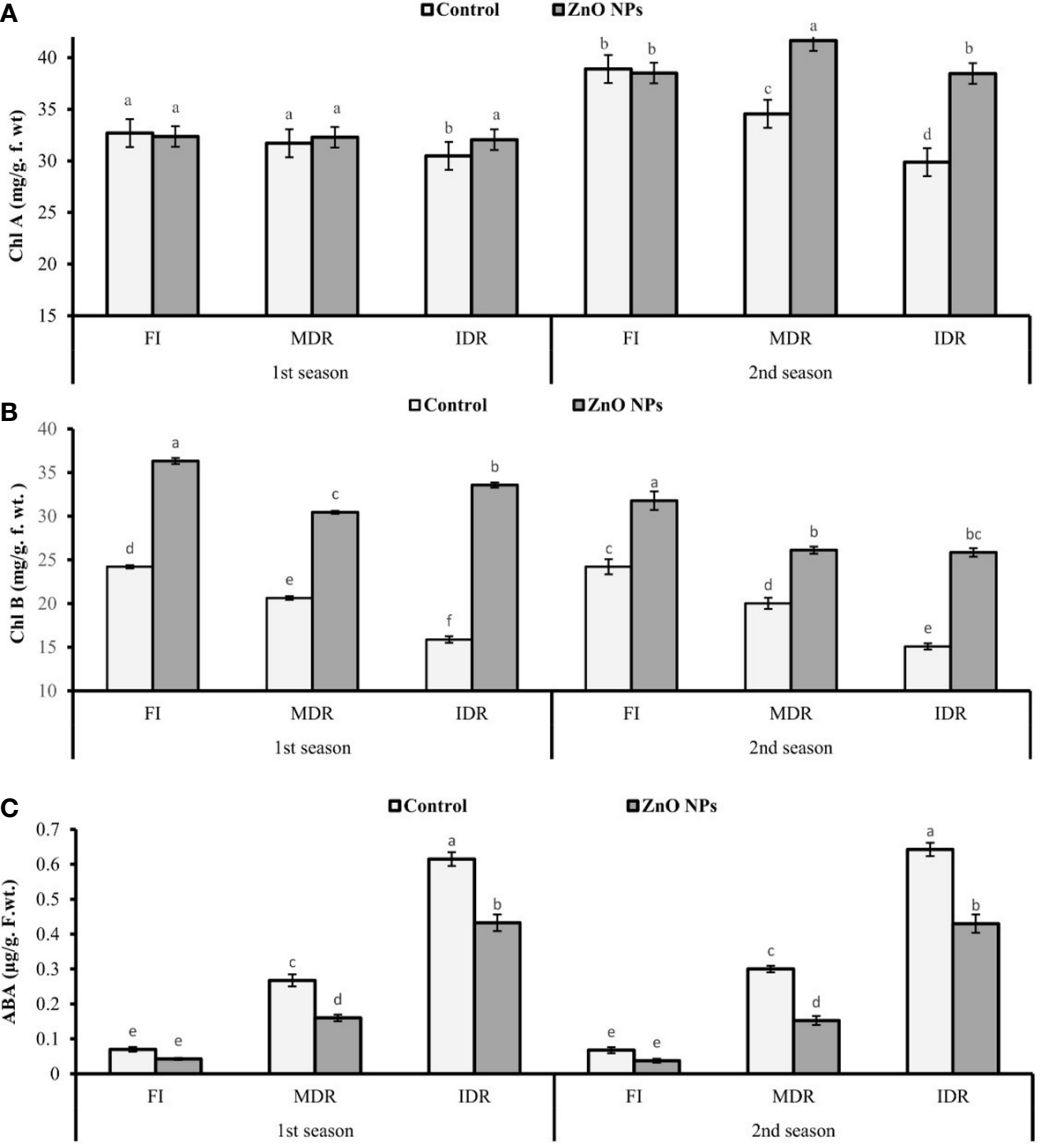
Effect of foliar application of ZnO nanoparticles (100 ppm) on **(A)** Chl a (chlorophyll a), **(B)** Chl b (chlorophyll b), and **(C)** abscisic acid content in the first and second seasons in full irrigation, moderate drought regimes, and intensive drought regime plants. The graph values are the mean ± SE of four replicates. Bars exhibited with different alphabets indicate the significant difference between samples by least significant difference (*p* ≤ 0.05).

The chlorophyll content, like chlorophyll a (Chl a) and chlorophyll b (Chl b), was examined under two regimes of drought MDR and IDR and well-watered (FI) plants. The plants of MDR showed a decrease of 3% (31.71 mg/g f. wt.) and 11% (34.56 mg/g f. wt.) in Chl a content in the first and the second season, respectively. However, IDR plants had a decreased Chl a content of 7% (30.49 mg/g f. wt.) and 23% (29.88 mg/g f. wt.) in the first and the second season, respectively, compared with FI plants as indicated in **Figure 2A**. Similarly, Chl b also decreased under drought stress. In the first season, Chl b was reduced to 15% (20.63 mg/g f. wt.) in MDR plants and 34% (15.88 mg/g f. wt.) in IDR in comparison with FI plants. The foliar application of ZnO NPs enhanced the concentration of Chl a to 2% (32.29 mg/g. f. wt.) and 5% (32.06 mg/g. f. wt.) in MDR and IDR plants in the first season, but in second season, the content of Chl a was increased to 21% (41.65 mg/g. f. wt.) in MDR plants and 29% (38.47 mg/g. f. wt.) in IDR plants compared with control plants (**Figure 2A**). Furthermore, improvement in Chl b content in MDR plants (48% and 30%) in both seasons was determined in the results when the plants were treated with foliar-applied ZnO NPs, while IDR plants showed a significant improvement of more than onefold and 41% in Chl b content in the first and the second season compared with untreated ZnO NP plants (**Figure 2B**).

### 3.3 Estimation of ABA

Data revealed that ABA significantly increased to twofold (0.27 µg/g. f. wt.) and fourfold (0.62 µg/g f. wt.) in MDR and IDR plants compared with well-watered plants (0.07 µg/g f. wt.) under drought regimes in the first season (**Figure 2C**). Similar results that showed a substantial increase in the concentration of ABA content to one-and-half-fold (0.30 g/g f. wt.) and more than fourfold (0.64 µg/g f. wt.) were obtained in the second season in MDR and IDR plants compared with control plants (0.07 µg/g f. wt.) in the second season. The foliar application of ZnO NPs lowered the content of ABA to 40% (0.16 µg/g f. wt.) and 30% (0.43 µg/g f. wt.) in MDR and IDR plants in the first season, but in the second season, the content of MDA was significantly reduced to 49% (0.15 µg/g f. wt.) in MDR plants and 33% (0.43 µg/g f. wt.) in IDR plants compared with control plants (**Figure 2C**).

### 3.4 Quantification of TSS, TPC, and TFC

**Figure 3:**
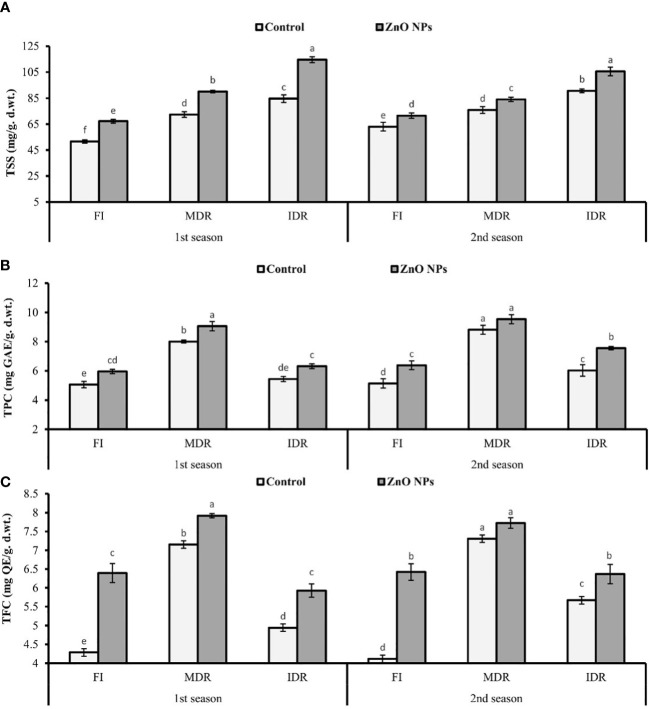
Effect of foliar application of ZnO nanoparticles (100 ppm) on **(A)** total soluble sugars, **(B)** total phenolic content, and **(C)** total flavonoid content in the first and second seasons in full irrigation, moderate drought regimes, and intensive drought regime plants. The graph values are the mean ± SE of four replicates. Bars exhibited with different alphabets indicate the significant difference between samples by least significant difference (*p* ≤ 0.05).

The presented results revealed that the concentration of TSS significantly increased to 40% (72.42 mg/g d. wt.) and 64% (84.69 mg/g d. wt.) in MDR and IDR plants compared with well-watered plants (51.60 mg/g d. wt.) under drought stress in the first season (**Figure 3A**). Similar results that showed a substantial increase in the concentration of TSS to 20% (75.90 mg/g d. wt.) and 44% (90.71 mg/g d. wt.) in MDR and IDR plants compared with control plants (63.05 mg/g d. wt.) were obtained in the second season. The foliar application of ZnO NPs enhanced the concentration of TSS to 24% (90.11 mg/g d. wt.) and 11% (84.09 mg/g d. wt.) in MDR plants of the first and the second season. The concentration of TSS was increased to 35% (114.59) and 16% (105.60 mg/g d. wt.) in IDR plants of both seasons compared with control plants (**Figure 3A**).

Under drought stress, TPC and TFC significantly increased to 58% (8.01 mg GAL Eq/g d. wt.) and 66% (7.15 mg QE/g d. wt.) in MDR plants (TRT5), while these were 8% (5.44 mg GAL Eq/g d. wt.) and 15% (4.94 mg QC Eq/g d. wt.) in IDR (TRT9) plants in the first season. A similar trend of significant increment in TPC and TFC was observed in MDR plants (77% and 72%) and IDR plants (17% and 37%) in the second season compared with fully irrigated plants (**Figures 3B, C**).

Under drought stress, ZnO NPs significantly enhanced the TPC to 13% (9.06 mg GAL Eq/g d. wt.) and 8% (9.54 mg GAL Eq/g d. wt.) in MDR plants (TRT7), while in severe drought stress (IDR), TPC considerably increased to 23% (6.32 mg GAL Eq/g d. wt.) and 25% (7.55 mg GAL Eq/g d. wt.) in plants compared with untreated ZnO NP plants in both consecutive seasons (**Figure 3A**). An increase of 20% (5.93 mg QC Eq/g d. wt.) and 12% (6.37 mg QC Eq/g d. wt.), respectively, was found in IDR plants, followed by 11% (7.92 mg QC Eq/g d. wt.) and 6% (7.73 mg QC Eq/g d. wt.) in MDR plants in the case of TFC amount compared with untreated ZnO NP plants in both consecutive seasons under drought stress regimes (**Figure 3C**).

### 3.5 Assessment of stomatal attributes

The stomatal attributes like stomatal density (SD), stomatal index (SI), stomatal length (SL), stomatal width (SW), pore length (PL), and pore width (PW) of abaxial and adaxial surface were measured to find out the effect of ZnO NPs (100 ppm) on the anatomical features and surface morphology of stomata under drought regimes as well as FI plants using micrographs.

**Figure 4:**
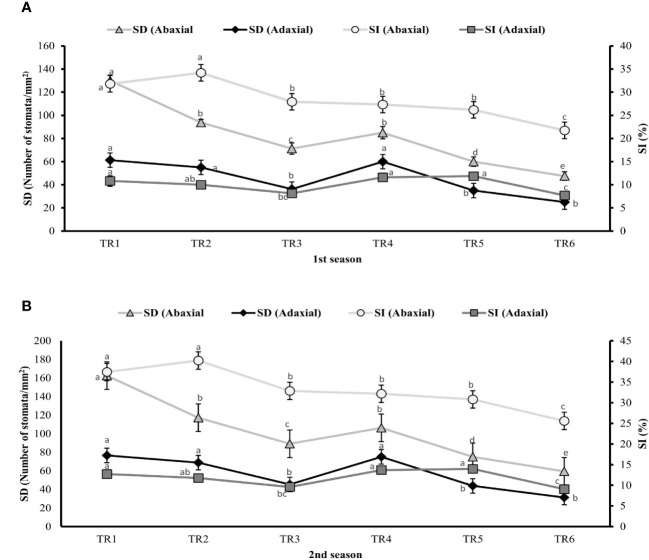
Effect of foliar application of ZnOx nanoparticles (NPs; 100 ppm) on stomatal density and stomatal index in the **(A)** first and **(B)** second seasons at different irrigation regimes. The graph values are the mean ± SE of four replicates. Bars exhibited with different alphabets indicate the significant difference between samples by least significant difference (p ≤ 0.05). TR1: FI, TR2: FI + ZnO NPs, TR3: MDR, TRT4: MDR + ZnO NPs, TRT5: IDR, and T6: IDR + ZnO NPs.

Plants of MDR treated with ZnO NPs showed a significant result by improving 19% (106 stomata/mm^2^) and 21% (100 stomata/mm^2^) of SD of abaxial surface in both seasons as indicated in TR4 (**Figure 4**) compared with untreated ZnO NP plants of MDR, while plants of IDR showed 21% and 23% decrease in SD of abaxial surface after post-anthesis in TR5 plants. Similarly, plants treated with ZnO NPs showed a significant increase of 67% (75 stomata/mm^2^) and 48% (67 stomata/mm^2^) in SD of adaxial surface of MDR plants in two consecutive seasons, while the plants of IDR showed low SD of adaxial surface when treated with ZnO NPs in TR6 compared with untreated ZnO NP plants in both seasons. The plants grown under drought regimes treated with ZnO NPs presented non-significant effects in SI of abaxial surface in TR4 plants and decreased the SI (abaxial) to 18% in IDR (TR5) in both the first and the second season compared with untreated ZnO NP plants. Plants treated with foliar-applied ZnO NPs showed a significant increase of 40% and 36% in SI of adaxial surface of TR4 in MDR plants in the first and the second season, respectively, while the plants of IDR (TR6) showed a decrease in SI (adaxial) in the first season and the second season compared with the control.

**Figure 5:**
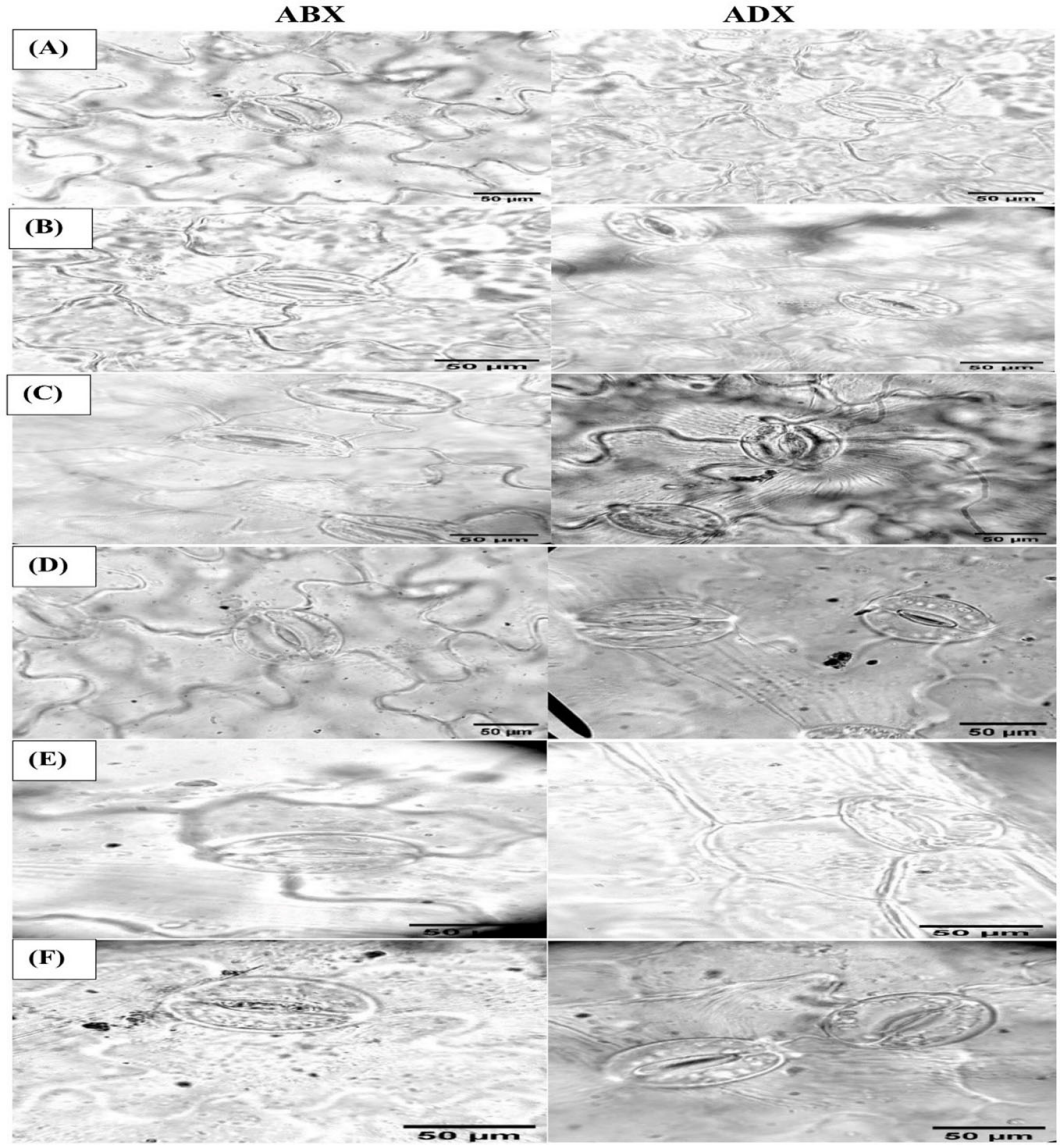
Microscopic magnitudes (×400) of the abaxial and adaxial surfaces of stomatal length, stomatal width, pore length, and pore width at different treatments: **(A)** TR1, **(B)** TR2, **(C)** TR3, **(D)** TR4, **(E)** TR5, and **(F)** TR6.

The stomatal dimensions clearly revealed that drought regimes decreased the SL and SW on both surfaces of leaves (abaxial and adaxial) as presented in **Table 3**, whereas the PL increased along with the application of moderate to intensive drought on abaxial surface, while non-significant results were observed on the adaxial surface. As for the concern related to PW, both surfaces showed a significant increment in MDR and IDR compared with FI plants. Foliar-applied ZnO NPs improve the SL and SW in FI and MDR plants, while IDR plants reduced the SL on abaxial surface. The adaxial surface showed a non-significant effect of ZnO NP application. In addition, foliar-applied ZnO NPs have no influential effects on PL and PW on the abaxial and adaxial surface, respectively, as indicated in **Table 3**. Micrographs of stomatal magnitudes were observed at ×400 as shown in **Figure 5**.

**Table 3:** Effect of different treatments on stomatal dimensions of abaxial and adaxial surface of *C. sativum* under drought regimes in the first and second seasons.

	SL (µm)		SW (µm)		PL (µm)		PW (µm)	
Treatments	ABX	ADX	ABX	ADX	ABX	ADX	ABX	ADX
First season								
TR1	28.5 ± 1.99d	31.1 ± 2.28b	21.3 ± 1.21b	24.9 ± 0.63a	14.9 ± 0.88cd	16.7 ± 1.22ab	4.29 ± 0.45b	3.76 ± 0.52d
TR2	30.9 ± 0.69cd	31.4 ± 0.75b	25.6 ± 0.83a	24.2 ± 1.04a	13.0 ± 1.26d	14.9 ± 1.02b	6.61 ± 0.50a	4.69 ± 0.81cd
TR3	37.7 ± 0.51a	33.5 ± 3.20ab	24.9 ± 1.06a	25.1 ± 0.81a	19.3 ± 1.35ab	16.0 ± 0.92ab	6.10 ± 0.52a	5.65 ± 0.22bc
TR4	34.6 ± 0.46b	38.5 ± 1.43a	26.6 ± 1.68a	26.4 ± 0.85a	21.2 ± 1.18a	19.8 ± 1.07a	6.94 ± 0.78a	9.24 ± 0.60a
TR5	38.8 ± 1.01a	31.8 ± 1.20b	26.6 ± 0.77a	27.06 ± 1.85a	16.8 ± 1.72bc	15.2 ± 1.23b	5.78 ± 0.58a	7.43 ± 0.44ab
TR6	32.8 ± 1.54bc	33.0 ± 3.34ab	23.8 ± 0.89ab	27.0 ± 1.28a	14.5 ± 1.32cd	15.1 ± 1.78b	6.16 ± 0.21a	7.13 ± 1.22b
Second season								
TR1	27.8 ± 2.31c	33.4 ± 2.07ab	22.1 ± 0.97c	23.5 ± 0.35bc	16.0 ± 0.91bc	18.6 ± 0.99a	3.92 ± 1.03b	5.25 ± 0.19c
TR2	32.5 ± 1.16bc	29.9 ± 1.61bc	21.9 ± 0.97c	22.3 ± 0.83c	13.5 ± 1.55c	13.5 ± 0.60b	5.96 ± 0.83a	5.56 ± 0.68bc
TR3	38.1 ± 3.84ab	36.8 ± 3.70a	25.7 ± 1.41b	26.3 ± 1.55ab	20.6 ± 1.37a	18.7 ± 1.69a	7.23 ± 0.12a	6.02 ± 0.47bc
TR4	39.2 ± 1.07a	33.2 ± 1.80ab	26.4 ± 0.89ab	25.3 ± 0.83abc	20.1 ± 1.86ab	16.1 ± 0.75ab	7.10 ± 0.78a	7.84 ± 0.55a
TR5	36.1 ± 1.89ab	34.2 ± 0.28ab	28.8 ± 1.60a	25.0 ± 1.33bc	15.8 ± 1.09c	14.6 ± 1.51b	6.38 ± 0.82a	4.93 ± 0.81c
TR6	35.6 ± 2.63ab	27.8 ± 0.74c	24.2 ± 0.75bc	28.6 ± 2.41a	17.0 ± 2.53abc	13.9 ± 0.99b	6.01 ± 0.33a	6.80 ± 0.62ab

SL, stomatal length; SW, stomatal width; PL, pore length; PW, pore width; ABX, abaxial; ADX, adaxial.

### 3.6 Interpretation of PCA

**Figure 6:**
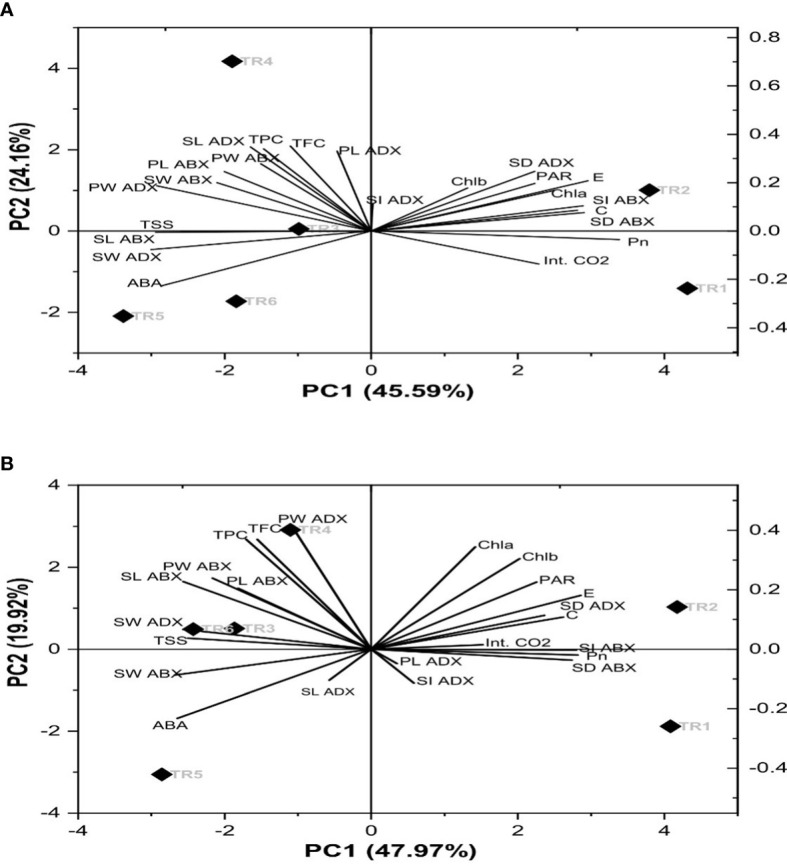
Principal component analysis of the chemical components of Essential oil under drought stress in two consecutive seasons. PC: principal component: **(A)** first season and **(B)** second season. TR, treatment; ABA, abscisic acid; Chla, chlorophyll a; Chlb, chlorophyll b; Pn, net photosynthesis; C, stomatal conductance; *E*, transpiration rate; Int. CO_2_, internal carbon dioxide; TSS, total soluble sugars; TFC, total flavonoid content; TPC, total phenolic content; SL, stomatal length; SW, stomatal width; PL, pore length; PW, pore width; ABX, abaxial; and ADX, adaxial.

A biplot demonstrated the combination of scree and loading plot with respect to six different treatments as indicated in **Figure 6**. The eigenvalues of correlation matrix indicates that there were four principal components (PC) considered to account for variance in the observed six treatments (imparted 94%) in both consecutive seasons. Concerning the extracted eigenvector values that disclosed the physiological, biochemical, and stomatal characteristics of PCs, in our study, 10 parameters in terms of the original set of 23 parameters showed significant loadings in determination of drought tolerance within six treatments. Thus, with reference to principal component analysis (PCA), PC1 and PC2 exhibited 46% and 24% variability within data in the first season and while 48% and 20% variability in the second season. Therefore, these two PCs explained 70% and 68% of variance. The coefficient correlation of PC1 and PC2 further revealed that nine chemical constituents, *i*.*e*., TSS, SW adaxial surface (ADX), SL abaxial surface (ABX), PW ABX, PL ABX, TPC, TFC, PW, and ADX, had a weak to moderate correlation. These parameters showed a significant role in the ZnO NP-induced drought tolerance. Out of these, Chla, Chlb, PAR, E, SD, ADX, C, Int. CO_2_, SI ADX, Pn, SD ABX, PL ADX, and SI ADX showed a positive direction, while the rest of the parameters showed a negative direction in both seasons as shown in **Figures 6A, B**.

## 4 Discussion

Nanoparticles are an effective tool for modulation crop production and sustainable agriculture in upgrading the plant mechanism under drought stress conditions (Dieleman et al., 2015). Although a lack of water input from rainfall is typically the primary cause of drought stress, the loss of water from soils due to evaporation, which is exacerbated by high temperature events, high light intensity, and dry wind, can exacerbate an already existing drought stress event (Salehi-Lisar and Bakhshayeshan-Agdam, 2016). Drought stress conditions are common because of global climate change across huge areas on a worldwide scale (Hasanuzzaman et al., 2013). In addition to drought, salt stress is regarded as a key cause of water shortage in plants (Mostofa et al., 2018). It was found that drought stress is the most adverse environmental factor that is mainly caused by temperature fluctuations, irradiance, and water scarcity, although drought stress exhibits chronic, inconspicuous influence and its multifaceted nature has a significant impact on physiological, biochemical, morphological, and molecular characteristics of *C. sativum*, with a negative impact on photosynthetic capacity (Xiong et al., 2022). Therefore, application of ZnO NPs is a better option to evolve various strategies for adaptations as well as make adjustments to cope with drought stress as presented in the current study (Seleiman et al., 2021).

The current study revealed that an appropriate amount of water supply is important for the growth and development of plants. However, a reduction in the water content of soil beyond 50% FC could significantly influence plant performance, grain yield, and quality due to drought stress (Khan et al., 2021). Furthermore, it was observed that water availability which is less than the optimal level in the rhizosphere inhibits plant development and hence plant nutrient intake as reported by previous studies (Elemike et al., 2019; Desoky et al., 2021). It could be due to the ability of plants to minimize resource consumption and regulate their growth to deal with severe environmental conditions such as drought (Kaur and Asthir, 2017). Our results revealed that modulation of stomatal conductance may play a vital role in the process of CO_2_ fixation and maintaining the relative water content during photosynthesis (Wankmüller and Carminati, 2022), while the distortion in signaling pathway and loss in turgor pressure occur as a result of stomatal closure due to vapor pressure under drought conditions (Jin et al., 2017). As a result, we observed the yellowing of leaves, leaf blistering, and stunted growth of plants (Cohen et al., 2021). Furthermore, the retarded growth in *C. sativum* plants under drought stress could restrict the cell expansion affected by water deficiency. Hence, to compensate for water shortage, stressed plants of *C. sativum* maintain their osmotic adjustment by increasing the amount of TSS, TPC, and TFC (Ghani et al., 2022). It was observed that the maximum PAR value plays a very vital role in enhancing the physiological features such as Pn, *C*, and *E* as compared with low PAR values as reported by Proietti et al. (2021). When plants of *C. sativum* were treated with water deficit regimes MDR and IDR, the SD and SI have been reduced to conserve water loss and maintain internal water balance (Ding et al., 2021). The DILP variety is unable to adapt to environmental circumstances for sustainable growth as a result of prevailing conditions due to poor water availability.

Moreover, the study revealed the role of TSS in drought tolerance through the modulation of membrane integrity (major component) and adjustment in the osmoregulation of plants (El Sabagh et al., 2019). The current study reported that foliar application of ZnO NPs significantly improved the amount of TSS under water deficit regimes, as shown in **Figure 3A**. Our results are consistent with the findings of Raeisi Sadati et al. (2022) and Khan et al. (2021). A higher amount of TSS under drought stress on the account of foliar application of ZnO NPs is a phenomenal adaptation of plants for osmotic adjustments (Sun et al., 2020). Therefore, through the accumulation of TSS in cells, it retains the RWC, stimulated the osmotic potential, and improved the drought tolerance in *C. sativum* (Sadak et al., 2020).

The increment in the concentration of Chlb on the account of foliar application of ZnO NPs in drought regimes is inconsistent with the findings of Gurmani et al. (2012) that describe the role of Zn in the enhancement of chlorophyll content in tomato. The same approach has been observed in another study in white lupin (*Lupinus termis*) (Latef et al., 2017). The reason behind this increase could be the role of ZnO in carbonic anhydrase (Zn metalloenzymes). This is a Zn derivative enzyme that facilitates the plants’ photosynthetic machinery for the efficient utilization of CO_2_ (Priester et al., 2017).

The data shown in **Figure 2C** indicate the substantial decrease in ABA amount in *C. sativum* when treated with ZnO NPs under both drought regimes. A noticeable reduction was observed in IR50 plants when treated with NPs (Khan et al., 2022). The possible phenomena may be the initiation of melatonin synthesis in *C. sativum* that improved the activity of antioxidant enzymes (Luo et al., 2022). This may ameliorate the drought tolerance through a reduction in ABA and increasing the osmo-protectants (proline and chlorophyll content) as described by Luying Sun et al. (2020). One of the other prospects is the inactivation of ABA produced under drought stress through the conjugation with soluble sugars, specifically glucose, to produce ABA-glucosyl ester as reported by Srivastava (2002). In addition, a previous report revealed the role of ZnO NPs in the facilitation of downregulation of ABA gene expression in strawberry (*Fragaria ananassa*), which further endorses our findings (Li et al., 2015). Our results showed an improvement in stomatal conductance when plants treated with ZnO NPs further confirmed the reduction in ABA content that plays a key role in stomatal operations under drought stress (Khan et al., 2022).

Bioactive compounds like phenols and flavonoids are actively produced under drought as secondary metabolites, as indicated in **Figure 2C** in our results (Zahir et al., 2014). Available reports revealed the improvement in phenol and flavonoid concentration on the account of foliar application of ZnO NPs. The improved concentration of phenols and flavonoids in IR75 when treated with 100 ppm ZnO NPs that has been reported in the current experiment is contradictory to the results of El-Zohri et al. (2021). This study showed that the foliar application of 100 mg/L of ZnO NPs decreased the phenolic content in tomato due to toxicity, or the results may be differ due to different plant types, cultivars, and climatic conditions, but a number of studies that supported our results are available (Javed et al., 2017). It may be due to the protective role of phenols and flavonoids that is produced by plants at the onset of foliar metal spraying, like ZnO NPs, as a defensive approach (Skórzy Ska-Polit et al., 2004). Furthermore, phenols act as metal-chelating agents, and ROS scavengers play an important role in retaining the homeostasis of cell (Phuyal et al., 2020; Kołton et al., 2022). Flavonoids may be used as flavonol peroxidase for the cleansing of H_2_O_2_ (ROS) under drought stress (Yamasaki et al., 1997). Therefore, a higher amount of phenols and flavonoids improves the antioxidant activity of *C. sativum* (Gharibi et al., 2016). We assumed that the higher concentration of TPC and TFC under the influence of ZnO NPs showed a mechanistic approach of *C. sativum* to tolerate drought stress.

The results showed that the foliar application of Zn enhanced stomatal magnitudes that could promote drought tolerance as described by Khan et al. (2004). It could be due to the involvement of Zn in the synthesis and regulation of various enzymes like carboxylases, alcohol dehydrogenase, and carboxypeptidase that enhanced cell division and expansion as described by Dimkpa et al. (2019), which improved the SL, SW, and PL. Furthermore, Zn maintained the concentration of K^+^ within the guard cell that maintained membrane integrity and improved the g_s_, which significantly improved the growth under a water deficit scenario (Khan et al., 2003).

Additional literature stated that these ZnO NPs increased the chlorophyll content, stomatal conductance, transpiration rate, and water use efficiency in drought conditions to improve photosynthesis and plant growth (López-Valdez et al., 2018; Alabdallah et al., 2021; Sun et al., 2021). This might be the result of the accumulation of secondary metabolites (TSS, TPC, and TFC) for osmotic adjustment under water shortage that improves the function of photosynthetic machinery as well as the overall yield of *C. sativum* (Ghasemi et al., 2017). In the end, we assumed that ZnO*_x_* NPs increased the tolerance of *C. sativum* against water stress by enhancing the stomatal attributes, which improved the photosynthetic rate to provide sustainable plant growth. The PCA revealed that TR2 and TR4 showed significant drought tolerance compared with other treatments. Therefore, the efficacy of 100 ppm ZnO NPs in the amelioration of stress tolerance may be used as a coping strategy against drought conditions for sustainable plant growth.

## 5 Conclusion

In this study, the foliar application of 100 ppm ZnO NPs play an effective role in the modulation of growth mechanism of *Coriander sativum* L. under two regimes of drought stress. The results showed that the foliar application of ZnO NPs improves the photosynthetic activity and chlorophyll content by regulating the Pn, *E*, and *C*. These NPs increased the amount total soluble sugars, total phenolics content, and total flavonoid content in drought stress regimes. The maximum stomatal density and stomatal index were observed in the abaxial surface (ABX) rather than the adaxial surface in foliar-supplemented ZnO NP plants. Furthermore, alterations in the dimensions of stomata like stomatal length, stomatal width, and pore length have been found as an adoptive strategy against drought regimes. These adaptive changes induced by ABA may be of significant importance for the survival and better growth of *Coriandrum sativum* with limited or no water supply. Therefore, these findings indicated that ABA-assisted drought tolerance is a prerequisite for plants to respond fully to drought. The PCA analysis revealed the efficacy of ZnO NP-induced drought tolerance in moderate and intensive stress regimes. Overall, the drought stress regime MDR showed better results in ZnO NP-treated plants. Our study assured the simplest approach to expose the efficacy of 100 ppm of ZnO NPs in the amelioration of drought tolerance in *Coriander sativum* by applying drought stress regimes for sustainable plant growth. To understand this complex phenomenon of tolerance though, ZnO NPs need to be evaluated further at the molecular and transcriptomic levels to explore the genome annotations with a future perspective of sustainable agriculture.

## Data availability statement

The original contributions presented in the study are included in the article/supplementary material. Further inquiries can be directed to the corresponding author.

## Author contributions

SA, MK, and YL contributed to the study concept and design and statistical analysis. SA, MK, AA, IU, QZ, and MM contributed to the analysis and interpretation of data. SA, MK and AN contributed to the investigation and resources. SA, MK, IU, AH, and QZ contributed to the drafting of the manuscript. MK, AA, MT, MA, and YL contributed to the review, editing, and proofreading of this manuscript. AN contributed to funding acquisition and study supervision. All authors contributed to the article and approved the submitted version.
